# Intramolecular Hydrogen Bond Improved Durability and Kinetics for Zinc-Organic Batteries

**DOI:** 10.1007/s40820-023-01263-7

**Published:** 2023-12-08

**Authors:** Tianjiang Sun, Jun Pan, Weijia Zhang, Xiaodi Jiang, Min Cheng, Zhengtai Zha, Hong Jin Fan, Zhanliang Tao

**Affiliations:** 1 Key Laboratory of Advanced Energy Materials Chemistry (Ministry of Education), Renewable Energy Conversion and Storage Center, College of Chemistry, Nankai University, Haihe Laboratory of Sustainable Chemical Transformations, Tianjin, 300071 People’s Republic of China; 2 School of Physical and Mathematical Sciences, Nanyang Technological University, Singapore, 637371 Singapore; 3 Shanghai Key Laboratory of Special Artificial Microstructure Materials and Technology, School of Physics Science and Engineering, Tongji University, Shanghai, 200092 People’s Republic of China

**Keywords:** Zn-organic batteries, H-PNADBQ polymer, Intramolecular hydrogen bond, Reduced solubility, Improved *π*-conjugated level

## Abstract

Organic compounds have the advantages of green sustainability and high designability, but their high solubility leads to poor durability of zinc-organic batteries. Herein, a high-performance quinone-based polymer (H-PNADBQ) material is designed by introducing an intramolecular hydrogen bonding (HB) strategy. The intramolecular HB (C=O⋯N–H) is formed in the reaction of 1,4-benzoquinone and 1,5-naphthalene diamine, which efficiently reduces the H-PNADBQ solubility and enhances its charge transfer in theory. In situ ultraviolet–visible analysis further reveals the insolubility of H-PNADBQ during the electrochemical cycles, enabling high durability at different current densities. Specifically, the H-PNADBQ electrode with high loading (10 mg cm^−2^) performs a long cycling life at 125 mA g^−1^ (> 290 cycles). The H-PNADBQ also shows high rate capability (137.1 mAh g^−1^ at 25 A g^−1^) due to significantly improved kinetics inducted by intramolecular HB. This work provides an efficient approach toward insoluble organic electrode materials.

**Supplementary Information:**

The online version contains supplementary material available at 10.1007/s40820-023-01263-7.

## Highlights

- Intramolecular hydrogen bond regulation is proposed to improve the quinone-based polymer (H-PNADBQ) solubility, conductivity, and kinetics.
- Intramolecular hydrogen bonds reduce molecular polarization and increase *π* conjugation level, thereby suppressing the dissolution of the H-PNADBQ and accelerating reaction kinetics of H^+^/Zn^2+^ uptake/removal.
- The H-PNADBQ electrodes exhibit excellent durability with high loading of 5 mg cm^−2^ and 10 mg cm^−2^, as well as high rate capability (137.1 mAh g^−1^ at 25 A g^−1^).

**Supplementary Information:**

The online version contains supplementary material available at 10.1007/s40820-023-01263-7.

## Graphical abstract

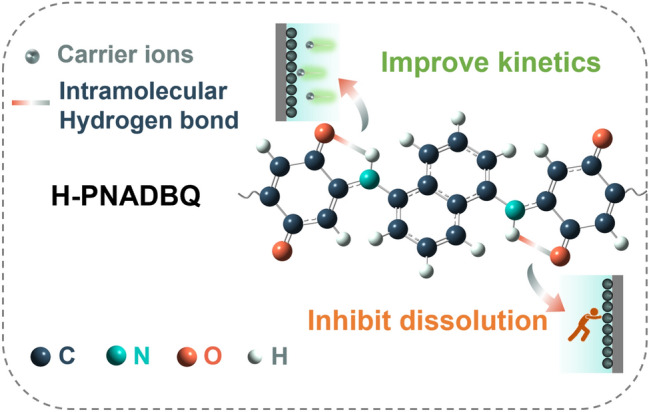

## Introduction

Selecting a suitable electrochemical energy system to store and convert renewable energy sources such as solar and wind energy is critical to achieve low-carbon goals [1, 2]. Among various electrochemical energy systems, aqueous zinc-ion batteries (AZIBs) are considered reliable alternatives for large-scale energy storage systems due to their environmental friendliness, simple assembly, and low cost [3–7]. As an important part, the cathode materials significantly affect the voltage, capacity, and durability of the AZIBs. Typical inorganic materials, such as manganese-based, vanadium-based, and Prussian blue analogs, have been widely reported in the past decades [8–15]. However, their poor structure stability and sluggish reaction kinetics limit practical application. Compared to inorganic materials, organic compounds have attracted extensive attention due to their flexible structural designability, tunable redox potential, and fast reaction kinetics dominated by pseudocapacitance [16–19]. But the main challenge is that the easy dissolution of organic materials in electrolytes leads to the unsatisfactory cycle life of AZIBs [20, 21]. Therefore, it is urgent to explore an effective strategy to hinder the dissolution of organic electrode materials.

Current strategies to reduce the solubility of organic cathodes include (1) coating with carbon materials; (2) lengthening the molecular chains; (3) extending the molecular planes [3, 22–25]. For example, Hu et al. developed poly(phenazine-alt-pyromellitic anhydride) with an extended *π* conjugation plane that exhibits fast ion diffusion [26]. Zhu et al. reported pyrazine-based porous aromatic frameworks/carbon nanotube composite with a high initial capacity of 328.5 mAh g^−1^ [27]. Although the material’s solubility is reduced to a certain extent, the complex process is still unsatisfactory. Modulating chemical bonds of organic compounds by molecular engineering design is simple and highly efficient. Particularly, hydrogen bond (HB) in host materials can affect their solubility in polar solutions [28, 29]. For instance, the hydrogen bonding network formed with carbonyl and amino groups on the tetra-amino-p-benzoquinone molecules allows good stability and facile proton conduction [30]. The hexaazatrinaphthalene-quinone with multiple hydrogen bonds (C–H⋯O) between the adjacent molecules in the plane shows low solubility in aqueous electrolytes [31]. Besides intermolecular HB, if the molecules can form intramolecular HB, the molecular solubility in polar solutions (for example, water solution) also will be reduced due to the saturability of HB. It can well explain that the solubility of *o*-diphenol is significantly lower than that of *p*-diphenol because intramolecular HB in *o*-diphenol can increase the symmetry of the molecules and reduce molecular polarization. Inspired by this, forming intramolecular HB, the solubility of solute molecules in polar solutions (such as aqueous solutions) also will decrease due to the saturability of HB [6–8]. Therefore, strategies to tune the intramolecular HB of organic electrode materials are expected to reduce their solubility.

Herein, abundant intramolecular HBs (C=O⋯H–N) are formed by the carbonyl group (C=O) in 1,4-benzoquinone (BQ) unit as HB acceptor with the HB-donor secondary amine groups (N–H) in 1,5-naphthalene diamine (1,5-NAD) unit. The introduction of intramolecular HBs in H-PNADBQ polymers not only suppresses the dissolution of active units by weakening the negative electrostatic potential of reactive sites and lowering the overall molecular polarization but also accelerates charge transfer by increasing the *π*-conjugated effect. In/ex situ ultraviolet–visible (UV–vis) spectroscopic tests revealed the low solubility of H-PNADBQ in 2 mol L^−1^ (M) Zn(CF_3_SO_3_)_2_ electrolyte. Benefiting from intramolecular HBs, the H-PNADBQ electrode with a high loading of 10 mg cm^−2^ can be stably cycled 290 times at 125 mA g^−1^ without capacity fading. Furthermore, the battery can achieve a high discharge capacity of 137.1 mAh g^−1^ even at a high current density of 25 A g^−1^, which is contributed by the high-capacitance-contribution kinetics. Meanwhile, a series of characterizations demonstrated the H^+^/Zn^2+^ co-adsorption ion-storage mechanism in the H-PNADBQ electrode.

## Experimental and Calculation

### Preparation of H-PNADBQ Material

**Scheme 1:**
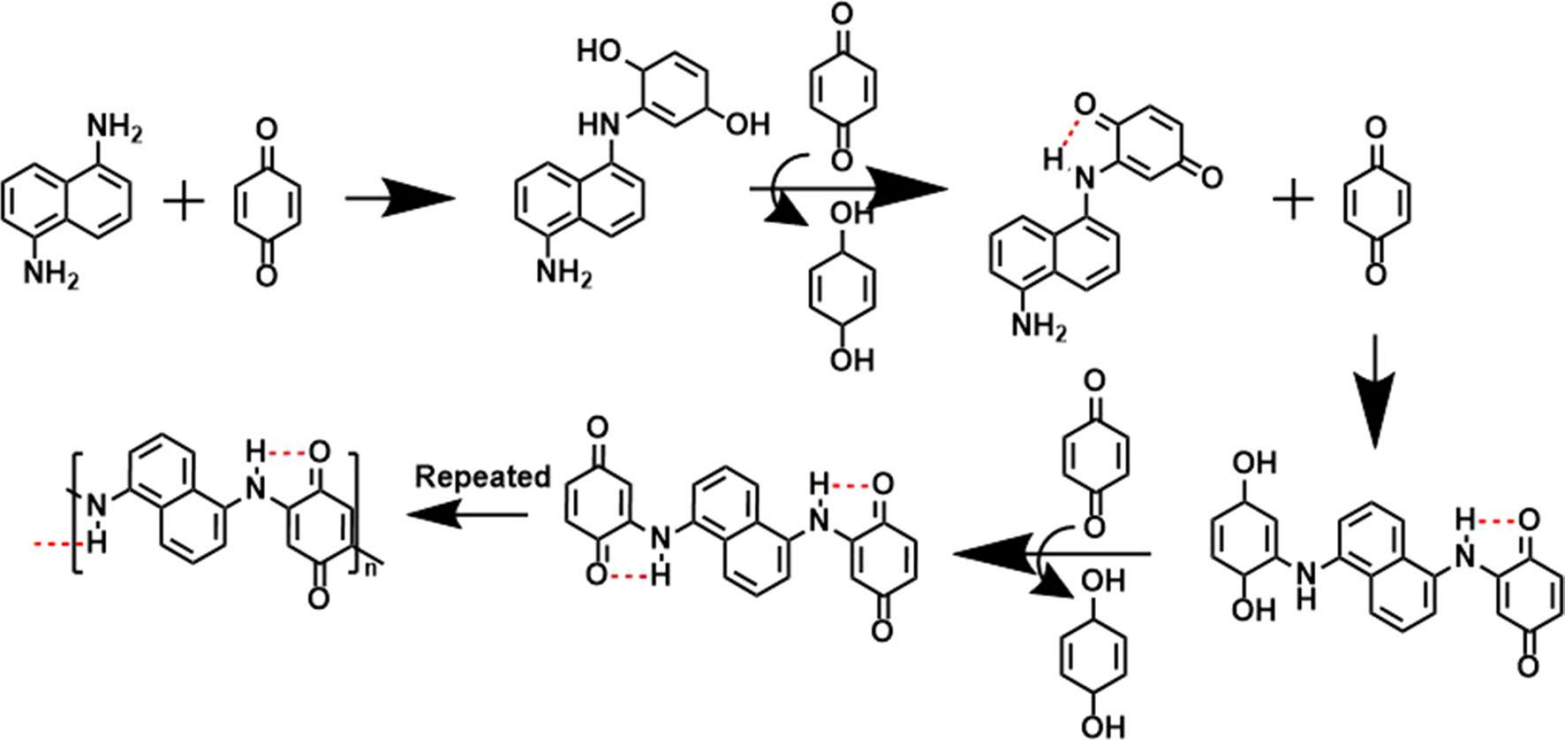
The polymerization reaction mechanism

The H-PNADBQ compound was prepared by a simple method (Scheme 1). In brief, 2 mmol 1, 5-NAD and 10 mmol BQ were added into a three-necked flask, and then 50 mL of ethanol was added to dissolve them. The mixture was heated and stirred at 70 °C for 5 h. After cooling to room temperature, the mixture was filtered and washed with anhydrous ethanol and ethyl acetate three times, respectively. The product was collected and dried under vacuum at 70 °C for 12 h to obtain a black power.

### Material Characterizations

The characterization of H-PNADBQ powers and electrodes was conducted by Fourier transform Infrared spectroscopy (FTIR, BRUKER TENSOR II (FTS6000)). The morphologies and microstructures of H-PNADBQ were tested by scanning electron microscopy (SEM, JEOLJSM-7500F) and Transmission electron microscope (TEM, Talos F200X G2). Powder X-ray diffraction (XRD) was collected over a wide 2*θ* range of 5°–60° (SmartLab 9 KW). The solubility of BQ and H-PNADBQ was evaluated by an ultraviolet–visible (UV–vis) spectrophotometer (UV-1900). The XPS spectra of Zn 2*p* were collected through PHI5000VersaProbe. The electrodes at the discharged and charged state were selected at the first cycle and were etched for 100 s before the XPS test. The thermostability analysis of H-PNADBQ was evaluated by a thermogravimetric analyzer with a heating rate of 5 °C min^−1^ (NETZSCH TG 209).

### Electrochemical Measurements

The slurry was prepared by mixing as-prepared H-PNADBQ powers, Ketjen black (KB), and polytetrafluoroethylene (PTFE) at an appropriate weight ratio of 7:2:1, and pressed onto stainless steel mesh (Φ 12 mm) to produce the H-PNADBQ electrodes. The electrodes were dried at 80 °C for 12 h under a vacuum. The mass loading of the H-PNADBQ compound was 2–10 mg cm^−2^. The batteries were assembled in 2032-type coin cells by using H-PNADBQ cathode, 2 M Zn(CF_3_SO_3_)_2_ electrolyte, Zn metal (0.05 mm, Φ 12 mm) anode, and glass fiber separator (Φ 16 mm). CV tests were carried out on an electrochemical workstation (CHI660E). The galvanostatic charge/discharge tests (0.2–1.4 V vs. Zn^2+^/Zn) were performed after resting for 3 h using a Neware battery test system (CT-4008, Shenzhen, China). The current density, specific capacity, specific energy density, and specific power density of the Zn//H-PNADBQ battery were calculated based on the active mass of H-PNADBQ. In the three-electrode system, Ag/AgCl and platinum plates were used as reference and counter electrodes, respectively. The theoretical specific capacity of H-PNADBQ polymer was calculated to be 183 mAh g^−1^ (the mole mass of repeated unit is 292 g mol^−1^ and the transfer electron number is 2).

### Computational Details

Density functional theory (DFT) calculations were performed using the Gaussian 16 program [32]. Geometry optimization and frequency analysis were performed in water solvents using the SMD solvation model. C, H, O, N use B3LYP functional and 6–31 + G (*d*, *p*) basis set. The calculation results of IRI and LOL-*π* were performed with Multiwfn 3.8 programs [33].

The intramolecular hydrogen-bond energy was predicted using the Atoms-in-molecules (AIM) theory, which follows Eq. (1) [34]:

1 $$E\left( {{\text{kcal}}\;{\text{mol}}^{{ - {1}}} } \right) = - {223}.0{8} \times \rho \left( {{\text{BCP}}} \right) + 0.{7423}$$

where *E* refers to hydrogen-bond energy. ρ(BCP) refers to the hydrogen-bond critical point, which can be obtained from Multiwfn 3.8 programs. Additionally, the intramolecular hydrogen-bond energy also could be evaluated by twisting molecular structure to break intramolecular hydrogen bonds (Fig. S3, PNADBQ without intramolecular hydrogen bond vs. H-PNADBQ with intramolecular hydrogen bond).

## Results and Discussion

### Design and Characterizations of H-PNADBQ

**Fig. 1:**
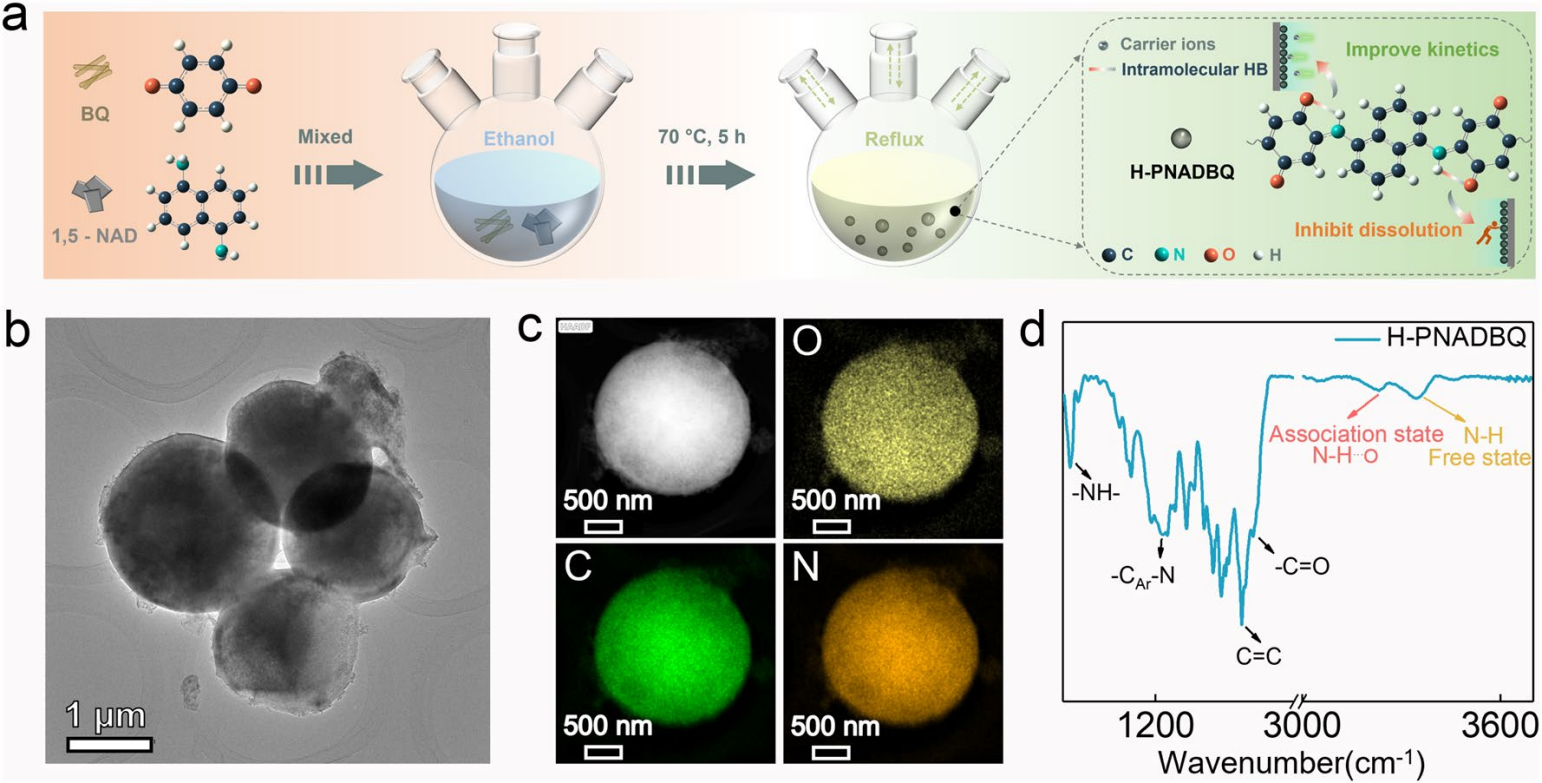
**a** Synthesis process of H-PNADBQ. **b** TEM image of H-PNADBQ. **c** TEM-mapping images of H-PNADBQ. **d** FTIR spectrum of H-PNADBQ

H-PNADBQ polymer is obtained from BQ monomer and 1,5-NAD under mild reaction conditions (Fig. 1a), which solves the problems of high sublimation and low conductivity of BQ [22, 30]. At the same time, this polymerization method is simple, convenient, and efficient, showing great potential for large-scale production. The as-prepared H-PNADBQ shows a spherical morphology with about a diameter of 2–3 µm (Figs. 1b and S1). Compared with the irregular morphology of BQ and 1,5-NAD, the spherical morphology of H-PNADBQ increases the contact area with the electrolyte. From the transmission electron microscope (TEM) mapping images in Fig. 1c, it can be seen that C, N, and O elements are uniformly distributed in the spherical structure. Fourier transform infrared spectroscopy (FTIR) was used to further explore the bonding characteristics of H-PNADBQ. The associated –NH– stretching vibration peak at 3232 cm^−1^ (N–H⋯O) and free –NH– stretching vibration peak at 3342 cm^−1^ (N–H) are distinguished. (Fig. 1d) [10]. The peak at 1580–1623 cm^−1^ corresponds to the stretching vibration of the C=O group. The C=O group in H-PNADPQ shows a significant red shift in comparison with that in BQ (Fig. S2), which can be attributed to the formation of the intramolecular HB (C=O⋯H–N) [35]. The thermogravimetric curve of H-PNADBQ was collected and shows a high decomposition temperature of 254 °C (Fig. S3).

### Theoretical Calculations and Solubility Testes of H-PNADBQ Molecule

**Fig. 2:**
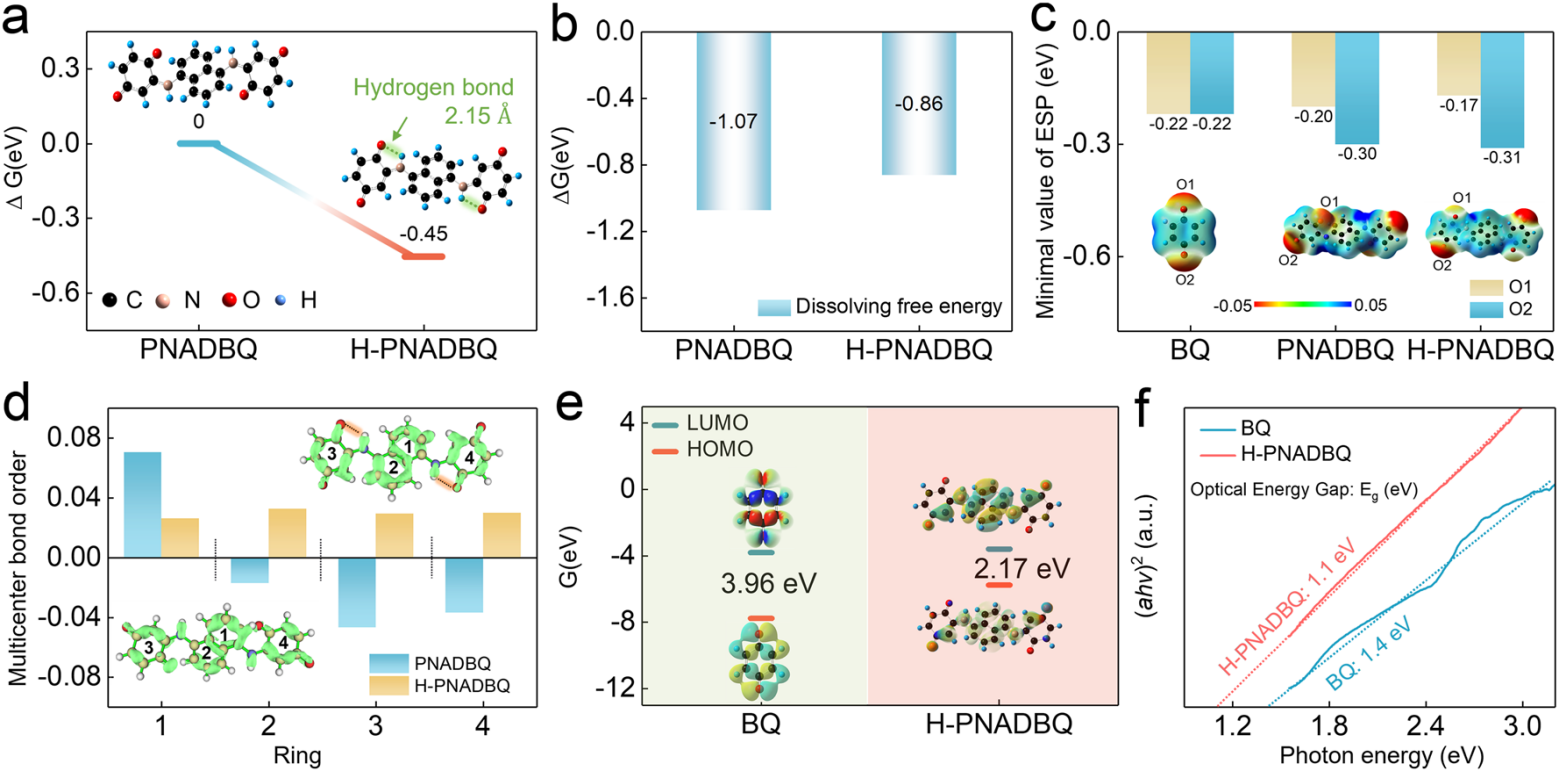
**a** Structural and energy optimization of PNADBQ and H-PNADBQ. **b** Dissolving free energy of PNADBQ and H-PNADBQ. **c** ESP images and ESP minima for different molecules. **d** LOL-*π* images and multicenter bond orders for PNADBQ and H-PNADBQ. **e** LUMO and HOMO plots of BQ and H-PNADBQ. **f** Optical energy gap of BQ and H-PNADBQ

Density functional theory (DFT) calculations were conducted to predict the effects of intramolecular HB in H-PNADBQ. A structural model was established as a control, namely PNADBQ, which cannot form intramolecular HB because the C=O and N–H groups are located on the opposite side (Fig. S4). The dihedral angle between the quinone ring and the naphthalene nucleus of the H-PNADBQ molecule is lower than that of PNADBQ (5.6° for H-PNADBQ vs. 41.8° for PNADBQ), suggesting a reduced molecular polarization and increased *π*-conjugated effect by the introduction of intramolecular HB. The H-PNADBQ shows a lower energy level compared with PNADBQ, indicating that the intramolecular HB stabilizes the structure of H-PNADBQ (Fig. 2a). The calculated intramolecular HB length in H-PNADBQ is 2.15 Å. Next, the intramolecular HB energy calculated using the Atoms-in-molecules theory is − 0.42 eV [12], which is close to the energy gap obtained by reversing the HB (− 0.45 eV (Fig. 2a), the calculation details in Computational Details). The short HB length and lower energy level allow the formation of C=O⋯H–N and it is classified as a strong HB. The HB interaction was visualized by the interaction region indicator (IRI), with the red region corresponding to strong interaction (Fig. S5) [36]. Due to the formation of intramolecular HB, the dipole moment of H-PNADBQ decreases (0.0025 D for PNADBQ vs. 0.0006 D for H-PNADBQ), which means that the molecular polarization of H-PNADBQ decreases. Therefore, the solubility of H-PNADBQ in aqueous solutions can be reduced [37]. The dissolution-free energy of H-PNADBQ is lower than that of PNADBQ, suggesting that the intramolecular HB can decrease the solubility of the host material (Fig. 2b). In addition, the molecular electrostatic potential (ESP) was performed to predict the reaction sites and activities of organic molecules (Fig. 2c). The negative ESP is concentrated on the C=O group, indicating that the C=O group has a high electrophilic tendency [38]. The difference is that the associated C=O group in H-PNADBQ (O1) showed more positive ESP than the free C=O group in PNADBQ (O1), and the ESP values in the O2 sites of PNADBQ and H-PNADBQ almost the same (Figs. 2c and S6). These results suggest that H-PNADBQ binds water molecules weaker than PNADBQ and is less soluble in water [39].

The influence of intramolecular HB on the *π*-conjugation effect was revealed using the localized orbital locator-*π* (LOL-*π*) method [40]. The *π* electron is better delocalized on different benzene rings in H-PNADBQ (Fig. 2d). The multicenter bond order was used to evaluate the level of the *π* conjugation effect. Except for benzene ring 1, the multicenter bond order of other benzene rings in H-PNADBQ is significantly higher than that of PNADBQ (Fig. 2d), indicating that H-PNADBQ has a better *π* conjugation effect. Hu et al. reveal that the extended *π* conjugation in poly(phenazine-alt-pyromellitic anhydride) accelerates the intramolecular electron transfer [26]. Thus, intramolecular HB can enhance the *π*-conjugation effect of the whole molecule, thereby facilitating charge transfer and suppressing dissolution [22, 26, 41, 42]. In addition, the small energy gap between the lowest unoccupied molecular orbital (LUMO) and the highest occupied molecular orbital (HOMO) of H-PNADBQ implies good electrical conductivity (Fig. 2e) [43, 44]. After H-PNADBQ accepted four electrons, the LUMO energy level increased and the structure remained unchanged in the HOMO plot, indicating that all C=O sites in H-PNADBQ could be reduced (Fig. S7) [45]. Benefiting from the improved *π* conjugation effect, H-PNADBQ shows a narrow optical energy gap of 1.1 eV, further revealing its good conductivity (Fig. 2f). DFT analyses indicate that the strong intramolecular HB can decrease H-PNADBQ polarization and hydrophilia and enhance its *π*-conjugation effect, thereby reducing solubility and improving the kinetics of H-PNADBQ in theory.

Ex/in situ UV–vis spectra were carried out to study the solubility of H-PNADBQ and BQ. H-PNADBQ power shows ultralow dissolution after resting in water for 72 h (Fig. S8). In contrast, BQ has a pronounced dissolution and a clear absorption peak around 290 nm. Then the solubility of both BQ and H-PNADBQ decreased in 2 M Zn(CF_3_SO_3_)_2_ electrolyte (Fig. S8). When in situ batteries were fabricated based on an H-PNADBQ cathode, Zn metal anode, and 2 M Zn(CF_3_SO_3_)_2_ or 2 M ZnSO_4_ electrolyte, a slight peak was found at about 290 nm in two electrolytes, and the intensity remained almost constant during the subsequent electrochemical process (Fig. S9a–d). By contrast, in situ UV spectra for BQ//Zn battery in 2 M Zn(CF_3_SO_3_)_2_ electrolyte exhibit an obvious absorption peak at about the 10^th^ spectrogram collection (Fig. S9e, f). These results provide sufficient evidence that the solubility of H-PNADBQ is significantly reduced by introducing intramolecular HB, which is an important guarantee for good cycle life.

### Electrochemical Performances of H-PNADBQ Electrodes

**Fig. 3:**
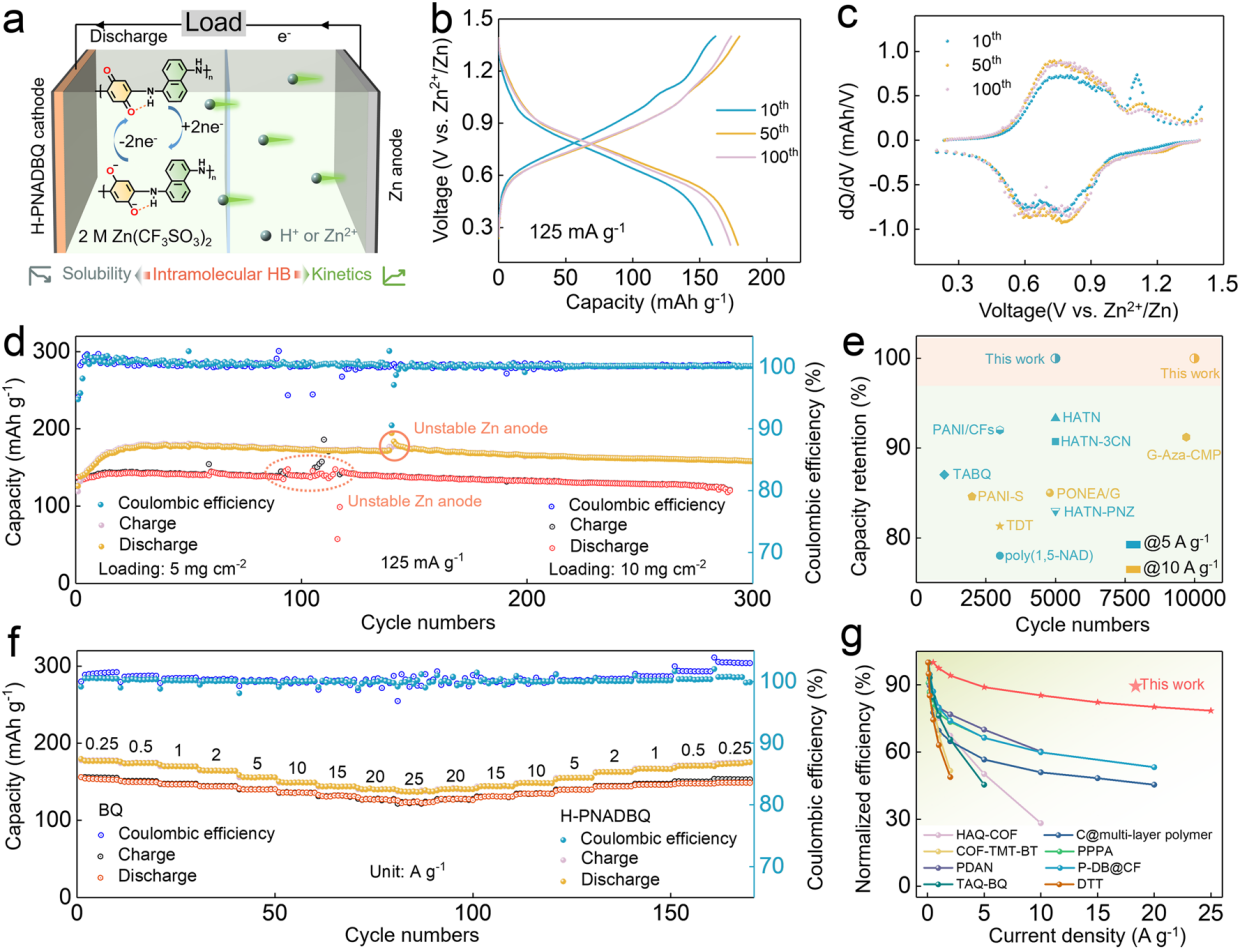
**a** Schematic of Zn//H-PNADBQ battery. **b** Charge–discharge curves of Zn//H-PNADBQ battery at 125 mA g^−1^. **c** dQ/dV plots of the Zn//H-PNADBQ battery. **d** Long cycling life of the Zn//H-PNADBQ battery at 125 mA g^−1^ with different H-PNADBQ loading. **e** Cycle performance comparison of Zn//H-PNADBQ battery to literature with other organic cathodes. **f** Rate performances of the Zn//H-PNADBQ and Zn//BQ batteries. **g** Rate performance comparison of Zn//H-PNADBQ battery to literature with other organic cathodes (the “normalized efficiency” means capacity retention based on the tested smallest current density)

The Zn//H-PNADBQ battery with 2 M Zn(CF_3_SO_3_)_2_ electrolyte was fabricated to investigate the effect of intramolecular HB on the electrochemical performance (Fig. 3a). The cyclic voltammetry (CV) curves of the Zn//H-PNADBQ battery in 2 M Zn(CF_3_SO_3_)_2_ electrolyte show two pairs of redox peaks, which is different from the Zn//BQ battery (Fig. S10). The nearly overlapping peak shapes indicate that H-PNADBQ has good electrochemical reversibility (Fig. S10a). The Zn//H-PNADBQ battery shows significantly decreased voltage polarization and increased discharge capacity after 50 cycles at 125 mA g^−1^ (Fig. 3b), corresponding to an activation process, which can be attributed to improved wettability between the electrode and the electrolyte and reduced impedance during cyclic processes (Fig. S11). It shows a high discharge capacity of 172.8 mAh g^−1^ after 100 cycles (Fig. 3b). The electrochemical stability of the H-PNADBQ electrode is also illustrated in the incremental capacity (dQ/dV) curves (Fig. 3c), i.e., with the same peak shape after 50 cycles. The cycling stability of the H-PNADBQ electrode with different active-material loadings was tested. These batteries obtain high discharged specific capacity at 125 mA g^−1^ after the activation process (176.2 mAh g^−1^ for ${\text{H - PNADBQ}}_{{{\text{(loading = 5}}\;{\text{mg}}\;{\text{cm}}^{{ - 2}} )}}$ vs. 140.2 mAh g^−1^ for ${\text{H - PNADBQ}}_{{{\text{(loading = 10}}\;{\text{mg}}\;{\text{cm}}^{{ - 2}} )}}$, the recorded data for 50th cycle), which results from the high electronic conductivity contributed by low LUMO–HOMO energy gap (Fig. 3d). These batteries can cycle over 290 times at 125 mA g^−1^ with high capacity retention, reflecting the low solubility induced by the intramolecular HB and practical application potential of H-PNADBQ (Fig. 3d). Specifically, the Zn anodes become unstable at low current density and deep charge/discharge, resulting in “hops” in the Coulombic efficiency of Zn//H-PNADBQ batteries (Figs. 3d and S12). In addition, the long-term cycle stability of the Zn//H-PNADBQ battery was also tested at 5 and 10 A g^−1^ and shows better durability than most of the organic cathode (Figs. S13a, b and 3e) [30, 39, 44, 46–52]. The voltage polarization increases only slightly after a long-time operation, indicating that the reaction kinetics of H-PNADBQ is relatively stable (Fig. S13c, d). While the BQ shows fast capacity decay in 2 M Zn(CF_3_SO_3_)_2_ electrolyte and a sharp increase in voltage polarization (Fig. S14). Additionally, the H-PNADBQ electrode also shows good electrochemical stability in 2 M ZnSO_4_ electrolyte (Fig. S15). The excellent cycling stability of H-PNADBQ in different electrolytes is inseparable from the effect of intramolecular HB on solubility.

The rate performance of the Zn//H-PNADBQ and Zn//BQ batteries was compared at different current densities from 0.25 to 25 A g^−1^, as shown in Figs. 3f and S16. The Zn//H-PNADBQ battery exhibits a discharge capacity of 137.1 mAh g^−1^ at an ultrahigh current density of 25 A g^−1^ and achieves a discharge capability of 78.5% at 0.5 A g^−1^ (Figs. 3f and S16a). By contrast, the BQ exhibits inferior rate capacity and Coulombic efficiency (Fig. 3f). The voltage plateaus of H-PNADBQ are well maintained even at high current density (Fig. S16b). It can achieve a high specific energy density of 108 Wh kg^−1^ at 19,625 W kg^−1^ (Fig. S17, calculated based on the mass of active H-PNADBQ). The excellent rate capability of H-PNADBQ may be related to the improved *π* conjugation effect by the intramolecular HB. Significantly, the rate capability of H-PNADBQ is superior to the reported organic materials (Fig. 3g) [21, 26, 43, 53–57].

### Kinetics and Reaction Mechanism Studies

**Fig. 4:**
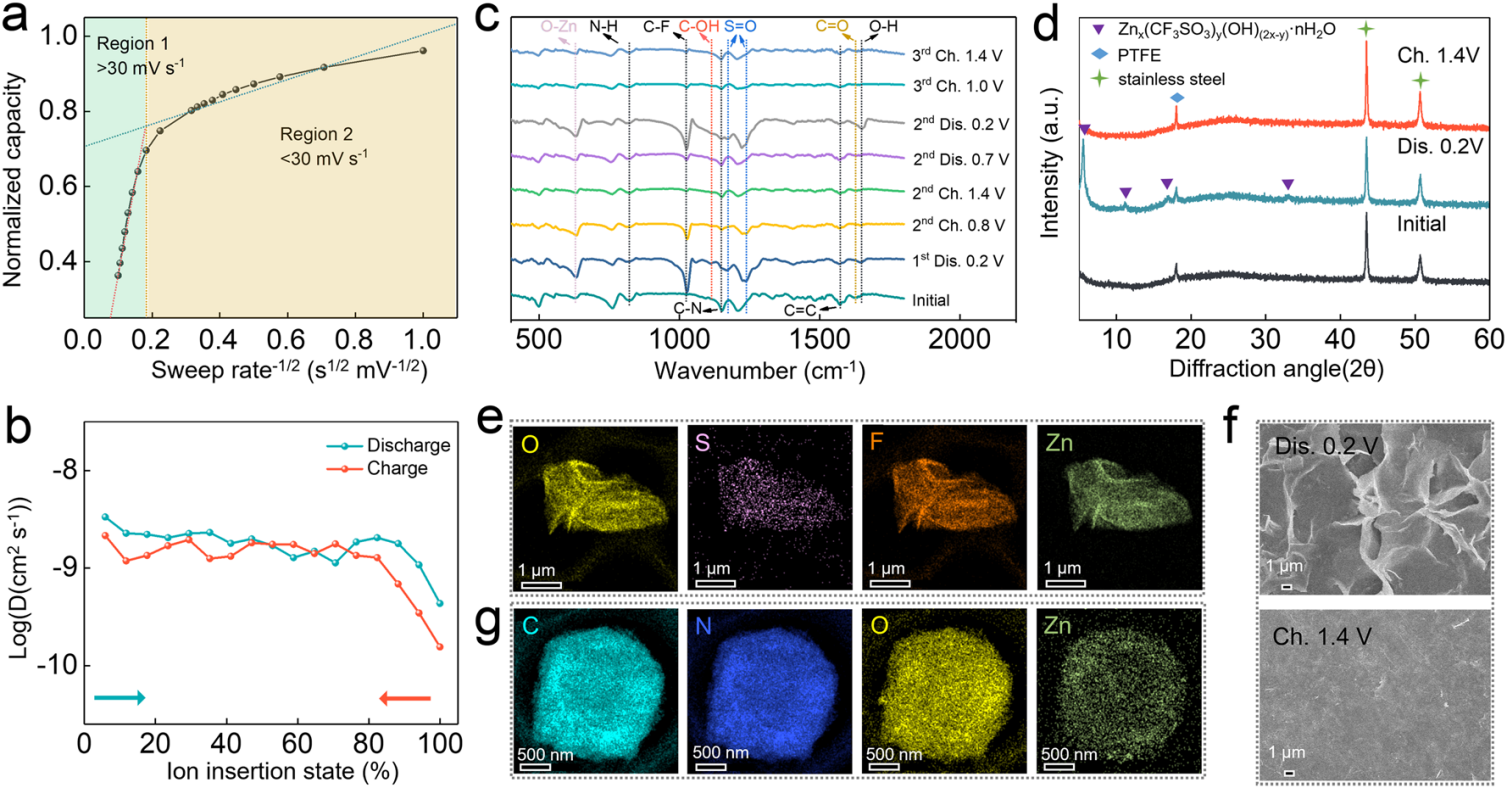
**a** Normalized capacity *vs.* scan rate^−1/2^ curves at scan rates of 1–100 mV s^−1^. **b** Calculated *D*_ions_ by the GITT test. **c** Ex situ FTIR and **d** XRD patterns of H-PNADBQ (Dis.: Discharged; Ch.: Charged). **e** TEM-mapping images of by-products at discharge state (0.2 V). **f** SEM images of H-PNADBQ at discharge (0.2 V) and charge state (1.4 V). **g** TEM-mapping images of H-PNADBQ at discharge state (0.2 V)

CV curves at different scan rates were conducted to investigate the charge storage kinetics of H-PNADBQ (Fig. S18a). The *b* values of peak 1 and peak 2 were 0.89 and 0.97 (Fig. S18b), respectively, fitted by a linear relationship between Log (peak current) and Log (scan rate), suggesting that H-PNADBQ displays a pseudocapacitance character [58, 59]. All capacitance contributions exceeded 80% at various scan rates from 1 to 5 mV s^−1^ (Fig. S18c, d), revealing the fast reaction kinetics of H-PNADBQ. The relationship between normalized capacity and scan rate was established to explore the rate-limiting step of the charge storage capacity (Figs. S19 and 4a) [60]. The relationship between normalized capacity and sweep rate is linear in Region 1, indicating that capacity is limited by diffusion contribution at high scan rates (30 < *v* < 100 mV s^−1^) (Fig. 4a). In contrast, there is no functional relationship between normalized capacity and scan rate in Region 2 (*v* < 30 mV s^−1^), revealing that the capacitive contribution limits the rate of charge storage (Fig. 4a). Furthermore, the ion diffusion coefficient was calculated by galvanostatic intermittent titration technique (GITT) to be 10^–10^ ~ 10^–8^ cm^2^ s^−1^ (Figs. S20 and 4b). Therefore, the capacitance-dominated charge storage behavior endows H-PNADBQ with fast reaction kinetics and thus a satisfactory rate performance.

The ion-storage mechanism of the H-PNADBQ electrode was investigated by various ex situ measurements. From the ex situ FTIR spectra (Fig. 4c), it can be seen that the peak at 1626 cm^−1^ attributed to the C=O group fades away gradually during the discharge, and then returns to the original state in the charged state, suggesting that the C=O group is the electroactive site of H-PNADBQ. Simultaneously, the peaks corresponding to the C–OH (1112 cm^−1^) and O–Zn (630 cm^−1^) signals appeared and disappeared reversibly, indicating that H^+^ and Zn^2+^ can be reversibly stored and released in H-PNADBQ [31, 61]. The simultaneous H^+^ and Zn^2+^ storage mechanism of H-PNADBQ was consolidated by controlling the cation type of the electrolyte (Figs. S21 and S22). In addition, new peaks corresponding to C-F (1028 cm^−1^), S=O (1174 and 1239 cm^−1^), and O–H (1650 cm^−1^) signals were detected during discharge and weakened during charge, which can be attributed to the formation and dissolution of by-product (Zn_*x*_(CF_3_SO_3_)_*y*_(OH)_(*2x−y*)_·nH_2_O) due to H^+^ uptake/removal (Fig. 4c) [62–64]. Commonly, the consumption of H^+^ produces excess OH^−^. The excess OH^−^ combines with ions to form Zn_*x*_(CF_3_SO_3_)_*y*_(OH)_(*2x−y*)_·nH_2_O by-products to maintain the electrically neutral environment of the electrolyte. Thus, the formation of by-products implies the H^+^-storage behavior of H-PNADBQ. The by-product formation is further revealed by the ex situ X-ray diffraction (XRD) patterns and TEM-EDS as well as SEM images (Figs. 4d–f and S23). The signals of Zn_*x*_(CF_3_SO_3_)_*y*_(OH)_(*2x−y*)_·nH_2_O were detected in the discharged state. This by-product displays a typical fold morphology and is absent in the charged state (Fig. 4f). Moreover, the spherical morphology of H-PNADBQ still is maintained in the discharged state (Fig. 4g), and the Zn element was detected on the discharged H-PNADBQ, further inferring that Zn^2+^ participated in the electrochemical reaction (Figs. 4g and S24). Additionally, the XPS pattern of Zn 2*p* in Fig. S25 also suggests the uptake/removal of Zn^2+^ in H-PNADBQ electrode at discharged and charged state. Thus, the H^+^/Zn^2+^ co-storage mechanism of H-PNADBQ was revealed by comprehensive characterizations.

## Conclusions

In summary, intramolecular HB is designed in H-PNADBQ polymer to improve its electrochemical performance. DFT calculations combined with various experiments showed that intramolecular HB plays a significant role in H-PNADBQ: (1) stabilizing the molecular structure; (2) suppressing the dissolution by reducing the ESP of reactive sites and molecular polarization; (3) improving the electronic conductivity and (4) accelerating the charge transfer by improving the *π*-conjugation effect. Benefiting from these effects, the H-PNADBQ with high mass loadings (5 and 10 mg cm^−2^) exhibits satisfactory durability and a high rate capacity (137.1 mAh g^−1^ at 25 A g^−1^). Moreover, H-PNADBQ exhibits fast reaction kinetics, which is mainly contributed by the ion-storage mechanism with high capacitive behavior. This work provides an efficient strategy for HB chemistry to design high-performance organic compounds for AZIBs.

## Supplementary Information

Below is the link to the electronic supplementary material.

Supplementary file1 (PDF 1758 KB)
