# Supplementary material for: Intramolecular Hydrogen Bond Improved Durability and Kinetics for Zinc-Organic Batteries
Source: Nanomicro Lett. 2023 Dec 8;16:46. doi: 10.1007/s40820-023-01263-7 (PMC10709292; doi:10.1007/s40820-023-01263-7)
Supplement: Supplementary file 1 — Supplementary file1 (PDF 1758 KB) [file 40820_2023_1263_MOESM1_ESM.pdf]

Supporting Information for

Intramolecular Hydrogen Bond Improved Durability and Kinetics for Zinc-Organic Batteries

Tianjiang Sun[1, 2], Jun Pan[2], Weijia Zhang[1], Xiaodi Jiang[2, 3], Min Cheng[1], Zhengtai Zha[1], Hong Jin Fan[2, *], Zhanliang Tao[1, *]

[1]Key Laboratory of Advanced Energy Materials Chemistry (Ministry of Education), Renewable Energy Conversion and Storage Center, College of Chemistry, Nankai University, Haihe Laboratory of Sustainable Chemical Transformations, Tianjin 300071, P. R. China

[2]School of Physical and Mathematical Sciences, Nanyang Technological University, Singapore 637371, Singapore

[3]Shanghai Key Laboratory of Special Artificial Microstructure Materials and Technology, School of Physics Science and Engineering, Tongji University, Shanghai 200092, P. R. China

*Corresponding authors. E-mail: fanhj@ntu.edu.sg (Hong Jin Fan), taozhl@nankai.edu.cn (Zhanliang Tao)

Supplementary Figures

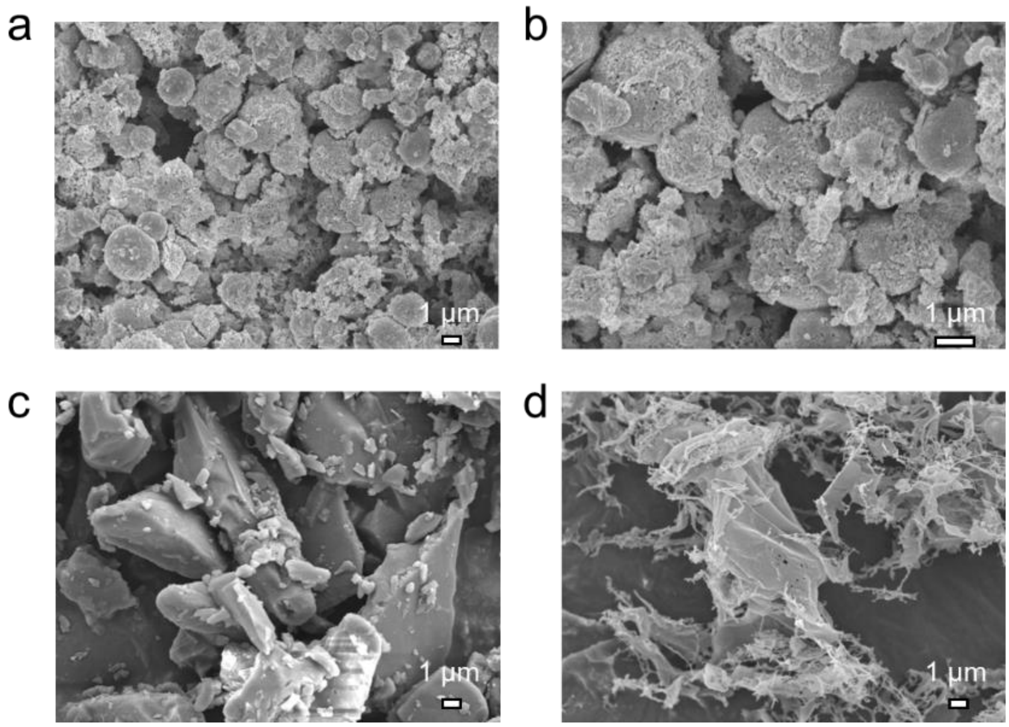

Fig. S1 SEM images of H-PNADBQ (**a, b**), 1, 5-NAD (**c**), and BQ (**d**)

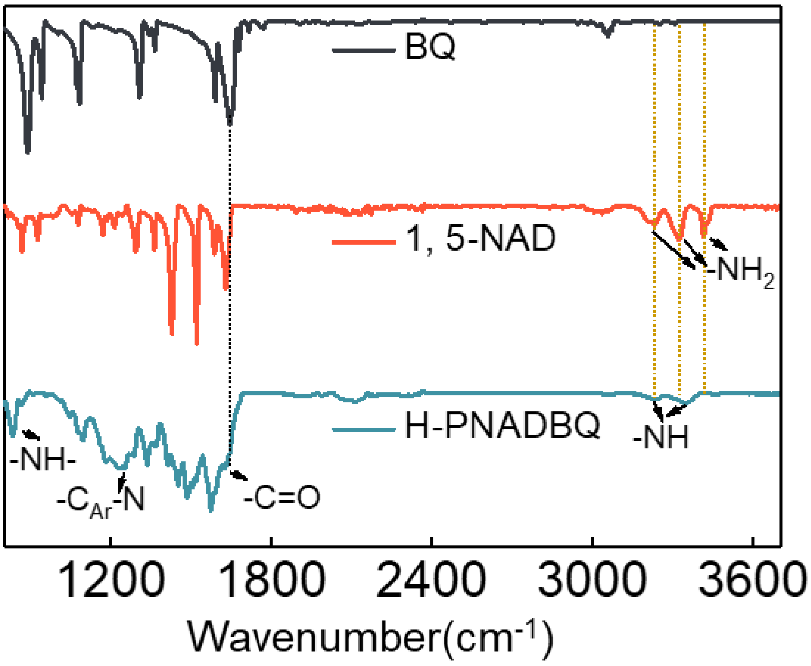

Fig. S2 FTIR of BQ, 1, 5-NAD, and H-PNADBQ

__**Note to Figure S2**__:

No peaks of -NH_2 are detected in H-PNADBQ, indicating successful polymerization. Besides, the peak of C=O in H-PNADBQ has obvious red shift in comparison with that in BQ, revealing decreased electron cloud density of O due to the formation of the intramolecular HB (C=O···H-N).

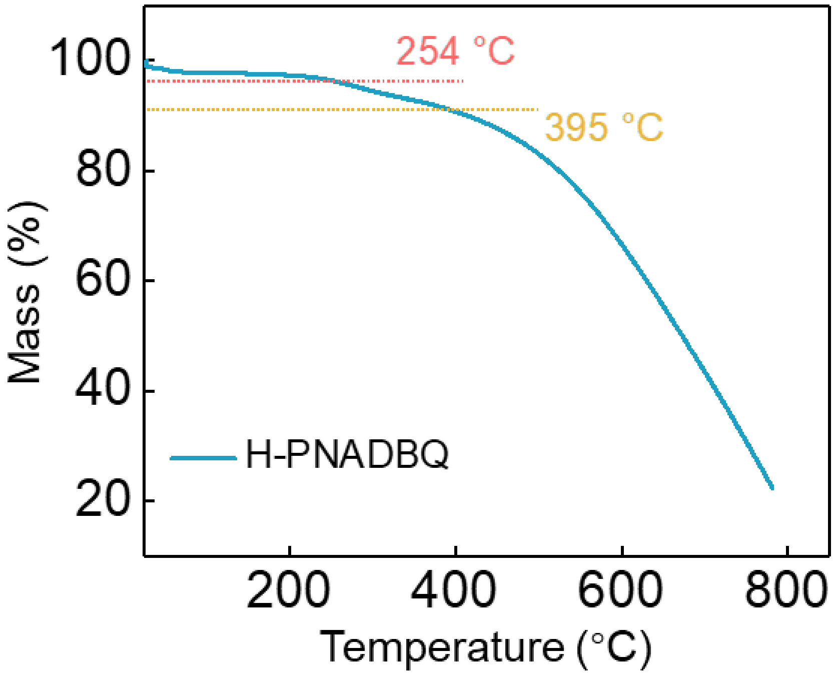

Fig. S3 Thermogravimetric curve of H-PNADBQ

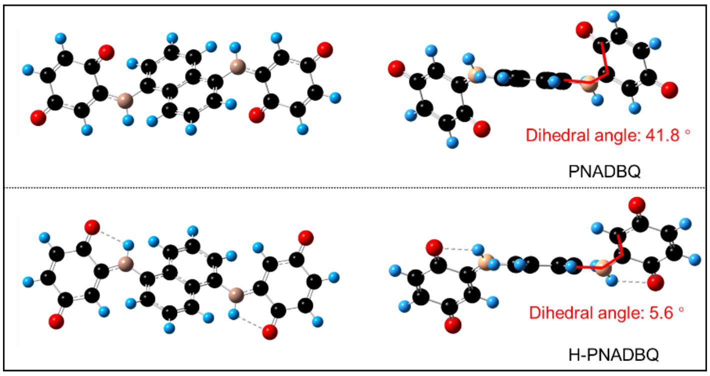

Fig. S4 Simulation structures of PNADBQ and H-PNADBQ

Note to Figure S4:

It can be seen from **Fig. S4** that the N-H and C=O groups are located in opposite sites in PNADBQ, so intramolecular HB cannot be formed. Here, PNADBQ was used as a control group to study the effect of intramolecular HB on polymer properties. Of note, the angle between the quinone ring and the naphthalene nucleus of H-PNADBQ molecule is lower than that of PNADBQ, suggesting a increased π-conjugated effect.

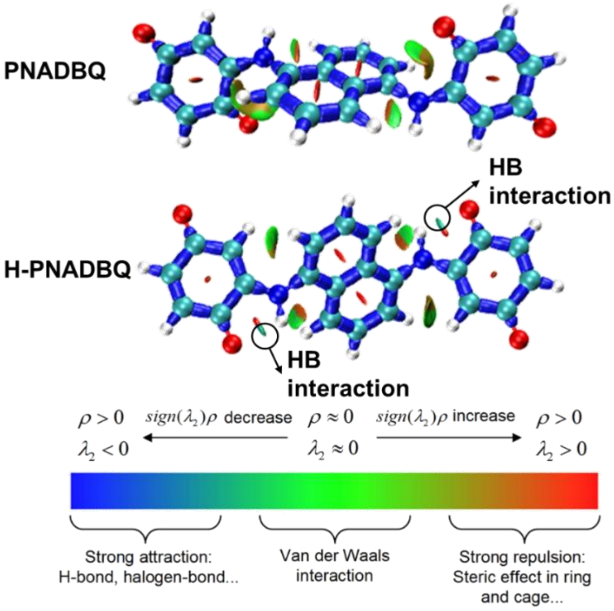

Fig. S5 IRI images of PNADBQ and H-PNADBQ

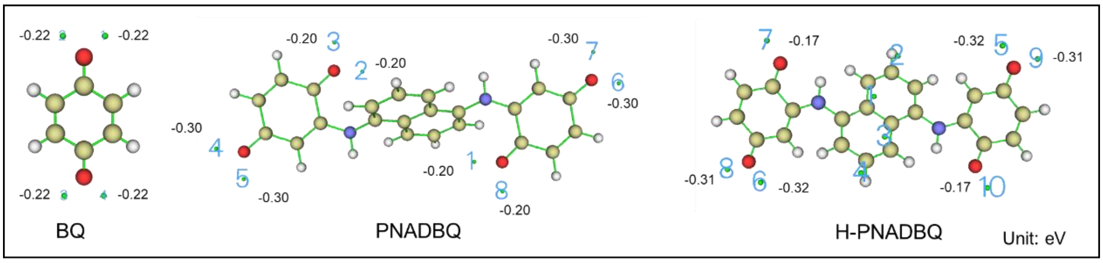

Fig. S6 ESP minimums for BQ, PNADBQ, and H-PNADBQ

Note to Figure S6:

Of note, the values in **Fig. S6** refer to the minimum ESP values on the C=O groups. As we know, the interaction strength between organic compounds and water molecules reflects the solubility of organic materials in water. The negative electron centers of organic materials can interact with H in water molecules. The more negative ESP of organic materials causes a stronger interaction with water, thereby promoting their dissolution. In this work, the C=O groups in H-PNADBQ show higher ESP than that in BQ and PNADBQ compounds, implying reduced solubility of H-PNADBQ due to the formation of intramolecular HB.

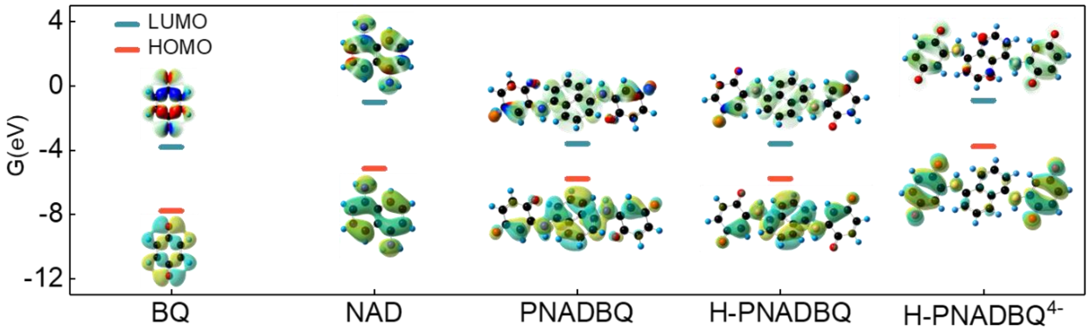

Fig. S7 LUMO and HOMO plots for different molecules

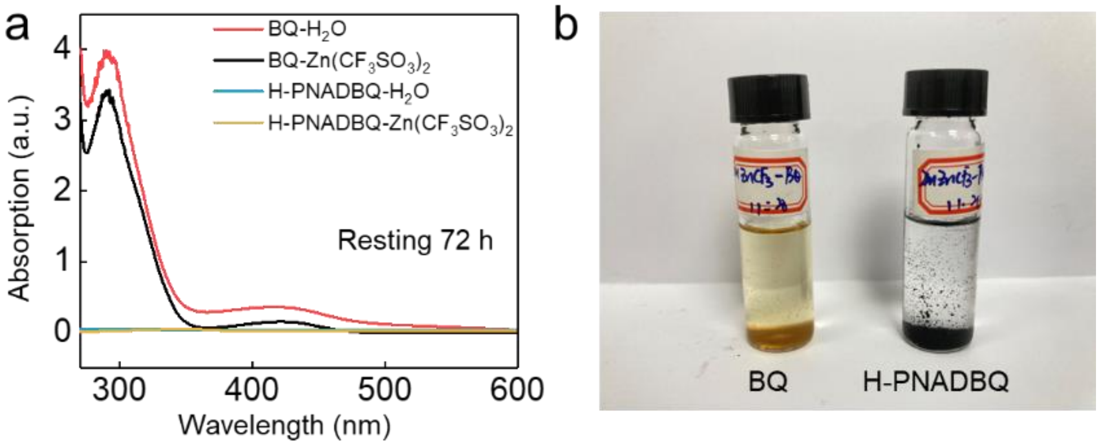

Fig. S8 a) UV-vis spectra of BQ and H-PNADBQ after standing in different solvents for 72 h. **b**) Optical photographs of these solutions after immersion

Note to Figure S8:

It can be seen from **Fig. S8** that BQ has an obvious absorption peak around 290 nm, and the solution is yellow, indicating that BQ has undergone violent dissolution. While no absorption peak is detected for H-PNADBQ and the solution still keeps clear, confirming the decreased solubility due to the existence of intramolecular HB.

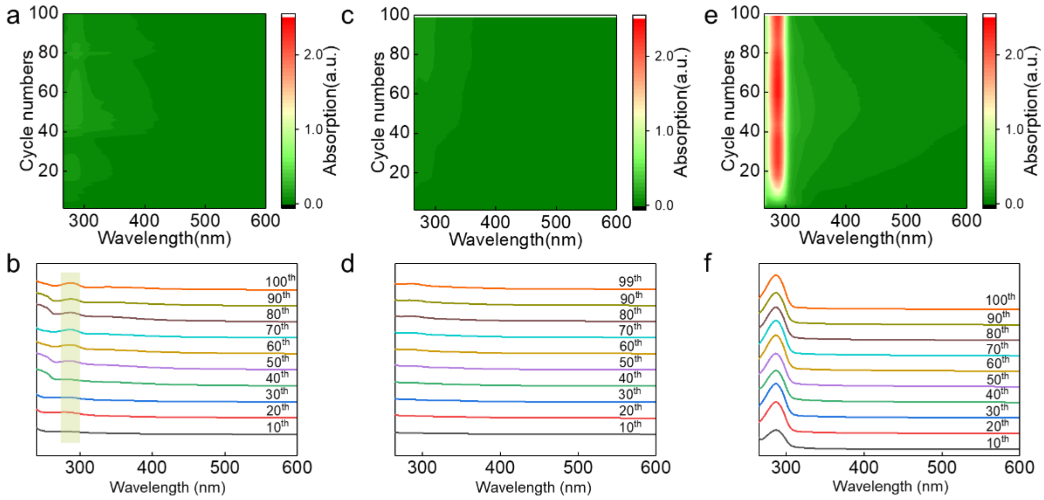

Fig. S9 In-situ UV-vis spectra of H-PNADBQ in 2 M $Zn(CF_3SO_3)_2$ electrolyte (**a, b**), H-PNADBQ in 2 M $ZnSO_4$ electrolyte (**c, d**), and BQ in 2 M $Zn(CF_3SO_3)_2$ electrolyte (**e, f**)

Note to Figure S9:

H-PNADBQ electrodes show a slight peak at about 290 nm when collected 50th spectrum in two electrolytes ($Zn(CF_3SO_3)_2$ and $ZnSO_4$ electrolytes). By contrast, the BQ electrode displays a clear adsorption peak when collected 10th spectrum in $Zn(CF_3SO_3)_2$ electrolytes, suggesting high solubility of BQ. Of note, $Zn(CF_3SO_3)_2$ salt shows better compatibility with Zn metal anode than $Zn(SO_4)_2$ salt. [S1] Therefore, we used 2 M $Zn(CF_3SO_3)_2$ solution as the electrolyte to study the H-PNADBQ electrochemical performances in this work.

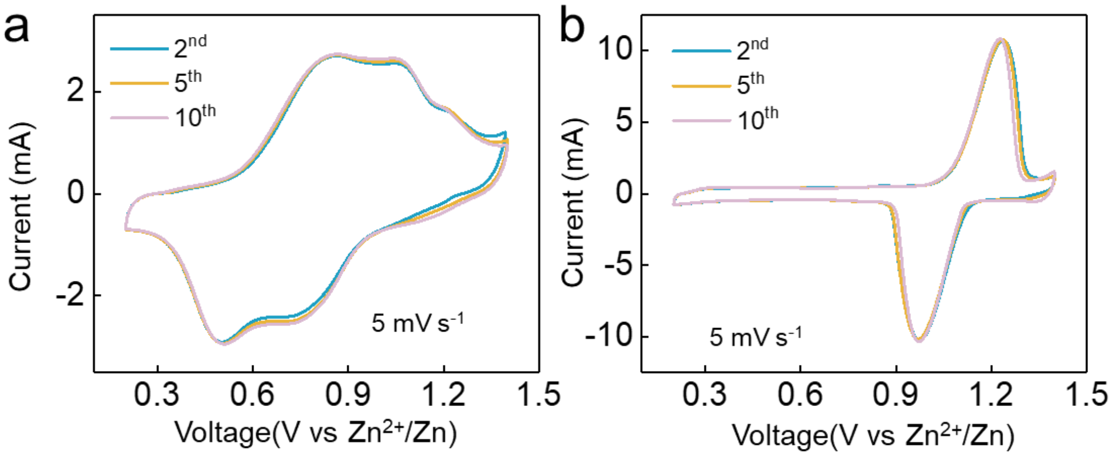

Fig. S10 a, b) CV curves of Zn//H-PNADBQ (**a**) and Zn//BQ (**b**) batteries at 5 mV s^{-1}

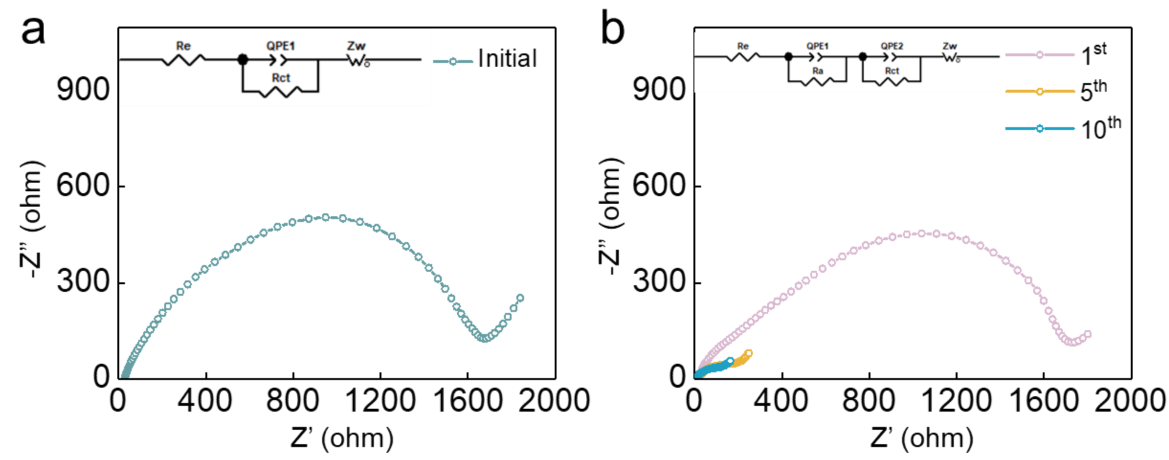

Fig. S11 The impedances of Zn//H-PNADBQ batteries at different states

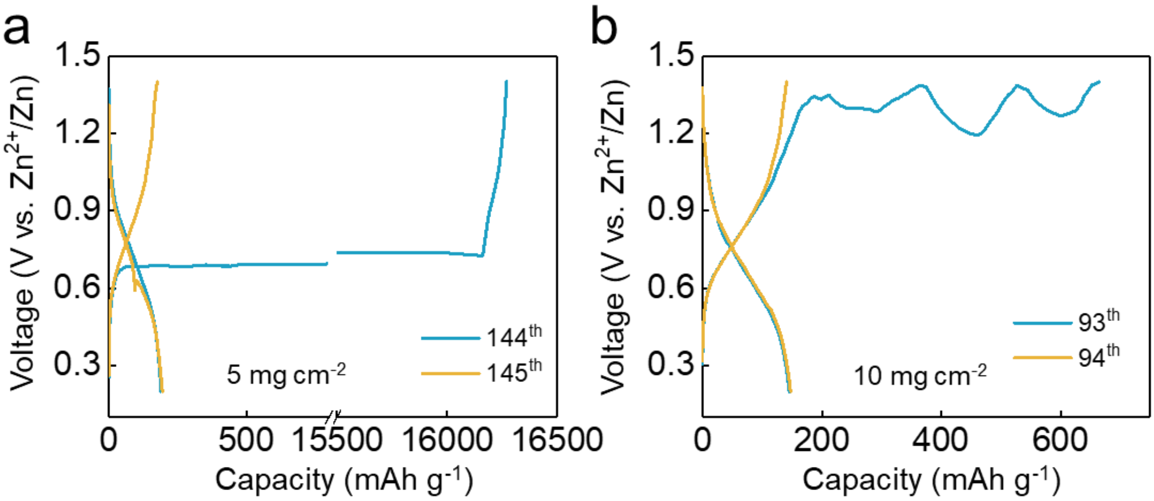

Fig. S12 Charge-discharge curves of Zn//H-PNADBQ with different loadings. **a**) 5 mg cm^{-2}. **b**) 10 mg cm^{-2}

<u>**Note to Figure S12**</u>:

At low current density, the deposition/stripping of the Zn anode may become more unstable.[S2] Additionally, the high-loading H-PNADBQ will consume more Zn from the Zn anode, which may lead to the Zn dendrite issue being more prominent.[S3] Both two factors may cause the batteries easily short out. A slight short circuit caused by Zn dendrite in the battery may result in the battery failing to charge. To evaluate the durability of the H-PNADBQ cathode, the failed Zn anode was replaced by a new Zn anode to enable the cell to work normally.

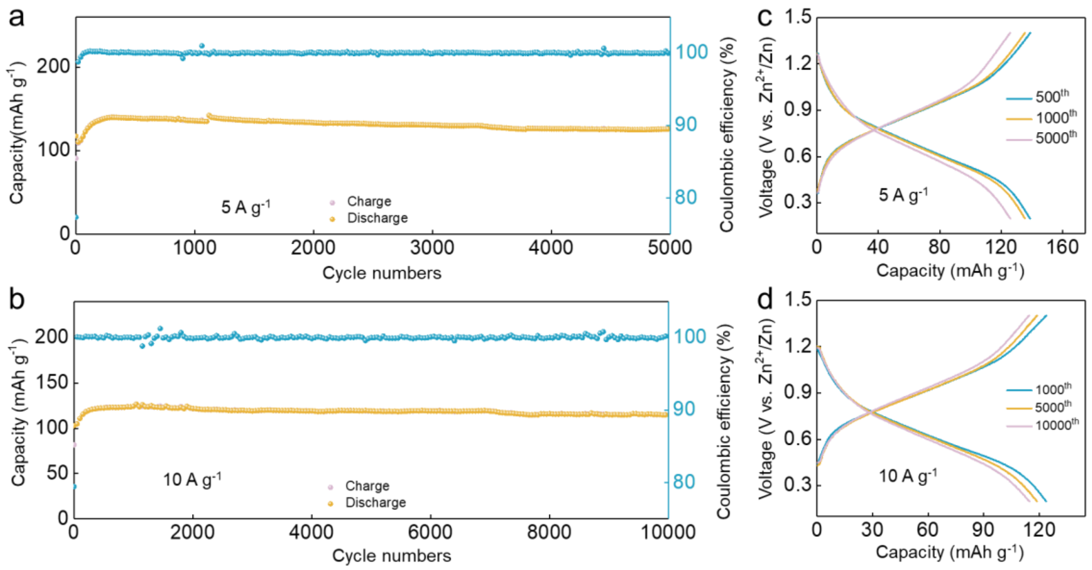

Fig. S13 Cycling stability of Zn//H-PNADBQ battery at 5 A g^{-1} (**a**) and 10 A g^{-1} (**b**). **c, d**) Charge-discharge curves of Zn//H-PNADBQ battery at 5 A g^{-1} (**c**) and 10 A g^{-1} (**d**)

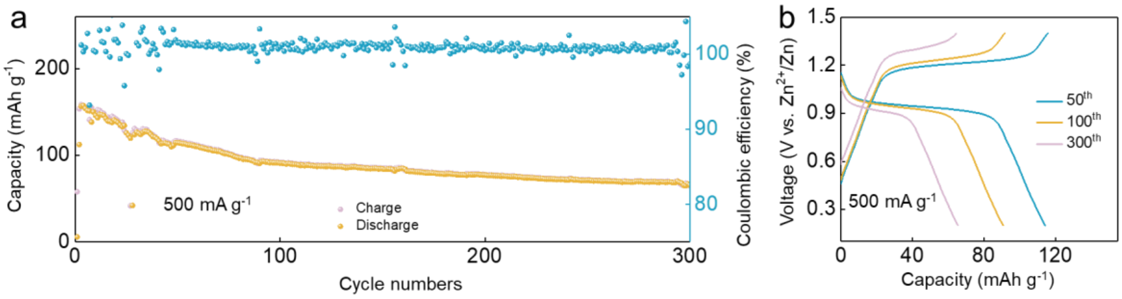

Fig. S14 Electrochemical performance of Zn//BQ battery at 500 mA g^{-1}. **a**) Cycling stability. **b**) Charge-discharge curves

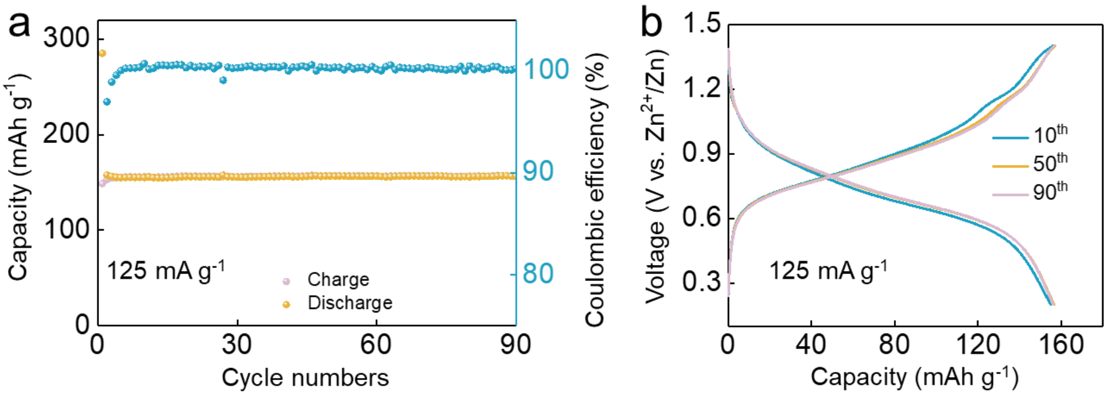

Fig. S15 Cyclic stability (**a**) and charge-discharge curves (**b**) of Zn//H-PNADBQ battery in 2 M $ZnSO_4$ electrolyte

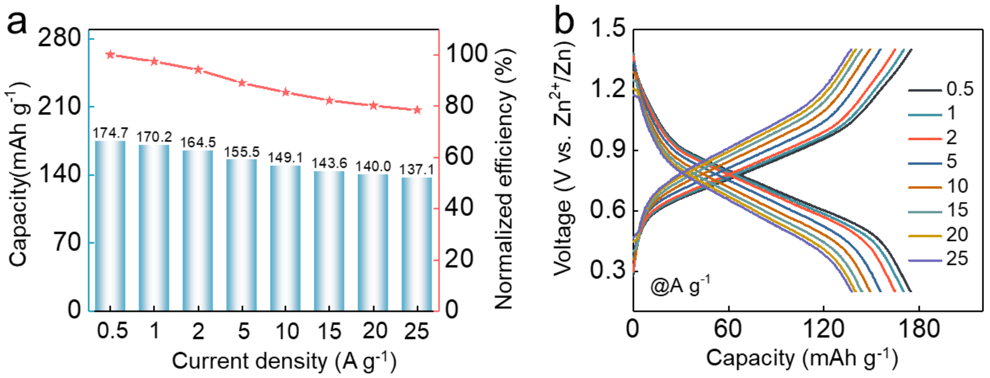

Fig. S16 a, b) Rate performance of Zn//H-PNADBQ battery at different current densities

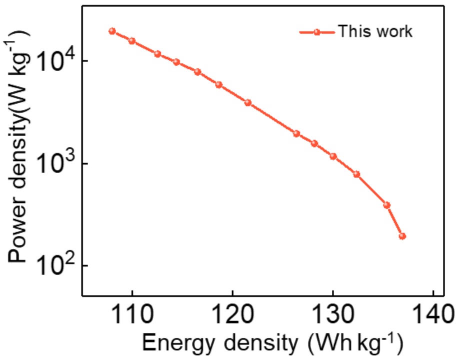

Fig. S17 Ragone plot of Zn//H-PNADBQ battery

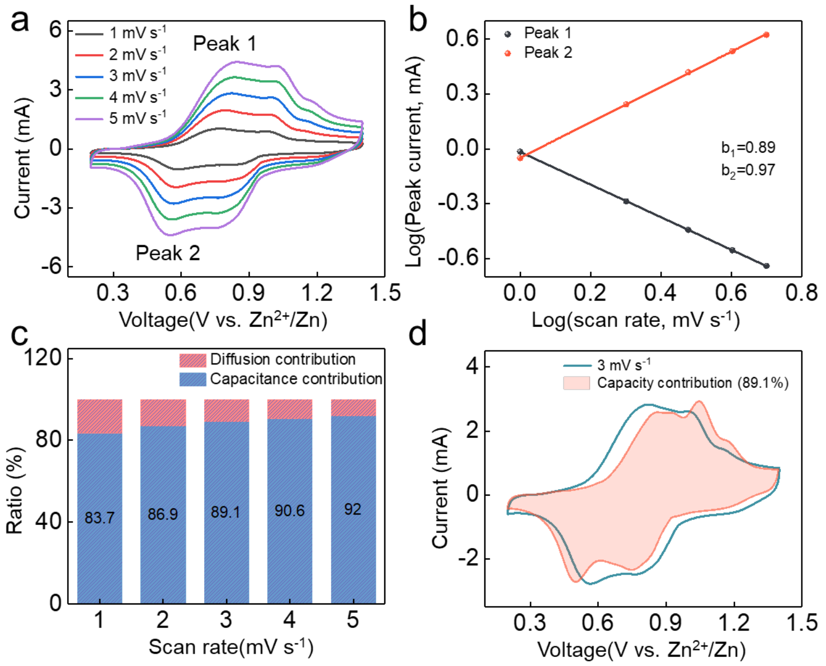

Fig. S18 a) CV curves of Zn//H-PNADBQ battery at different scan rates. **b**) Calculated *b* values for peak 1 and peak 2. **c**) Calculated ratio of capacitance contribution at different scan rates. **d**) Capacitance contribution ratio at 3 mV s^{-1}

Note to Figure S18:

To distinguish the type of charge transfer kinetics, the *b* values can be fitted by equation (S2): [S4]

$$i = av^b \tag{S2}$$

where *i* is the peak current (mA), and *v* is the scan rate (mV s^{-1}). The *b* values can be fitted by a linear relationship of log(*i*) = *b*log(*v*) + log(*a*).

The ratio of capacitance contribution and diffusion contribution is calculated according to equations (S3) and (S4): [S5]

$$i = k_1v + k_2v^{\frac{1}{2}} \tag{S3}$$

$$\frac{i}{v^{\frac{1}{2}}} = k_1v^{\frac{1}{2}} + k_2 \tag{S4}$$

where k_1v refers to the current part contributed by capacitance, and $k_2v^{\frac{1}{2}}$ refers to the current part contributed by diffusion.

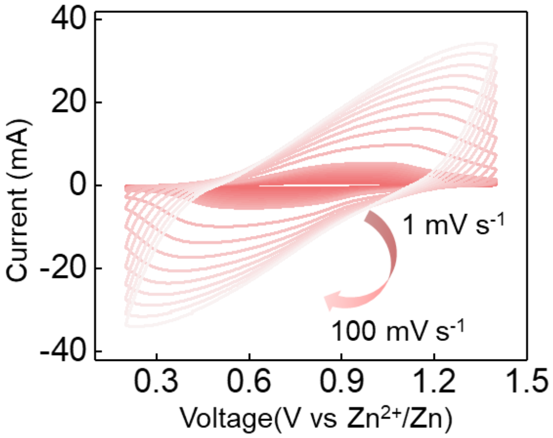

Fig. S19 CV curves of Zn//H-PNADBQ battery from 1 to 100 mV s^{-1}

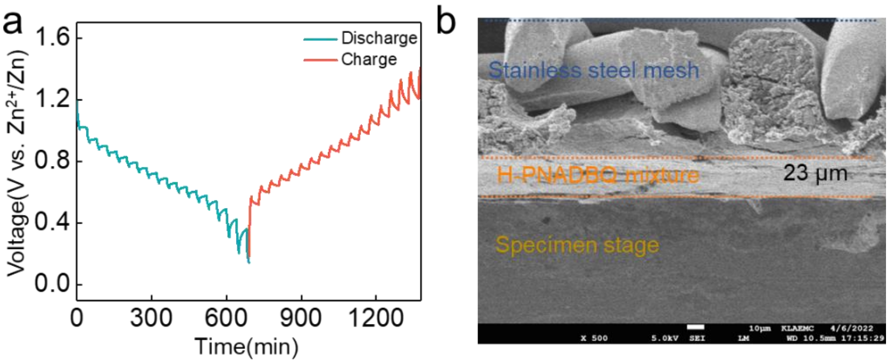

Fig. S20 **a**) GITT test of Zn//H-PNADBQ battery. **b**) The thickness of active material

Note to Figure S20:

The ion diffusion coefficient (D) is calculated by galvanostatic intermittent titration technique (GITT) measurement according to the following equation (S5): [S6]

$$D = \frac{4L^2}{\pi\tau}\left(\frac{\Delta E_S}{\Delta E_t}\right)^2 \tag{S5}$$

Where τ is the relaxation time. ΔE_s is the steady-state voltage change after the current pulse. ΔE_t is the voltage change during the relaxation process. L is the ion diffusion length (cm), approximately equal to the thickness of active material (23 μm).

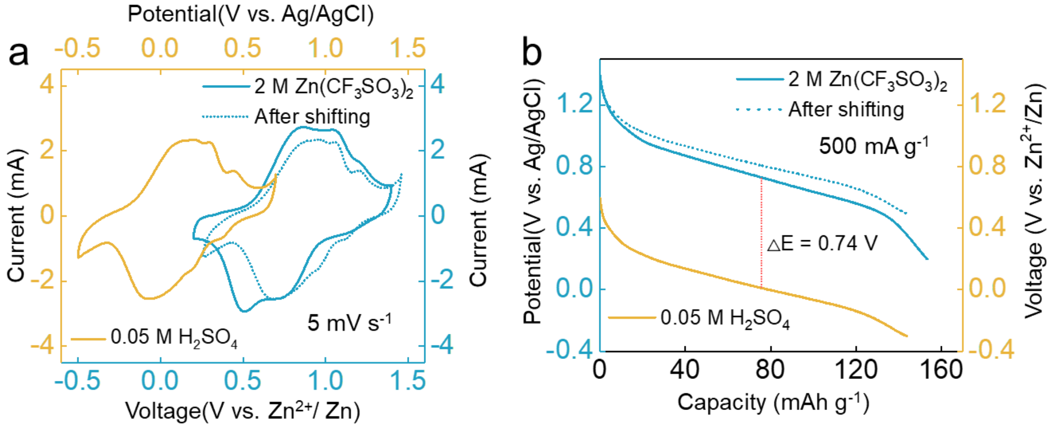

Fig. S21 a) CV curves of H-PNADBQ at different electrolytes. **b**) Charge-discharge curves of H-PNADBQ in different electrolytes (500 mA g^{-1})

Note to Figure S21:

The H^+-storage ability of H-PNADBQ was verified using 0.05 M H_2SO_4 electrolyte (pH=1) (**Fig. S21**). A three-electrode system with Ag/AgCl reference electrode, Pt counter electrode, and H-PNADBQ work electrode is fabricated. As shown in **Fig. S21a**, the shapes of the CV curves of the two electrolytes are almost the same. And a voltage shift of about 0.74 V is detected (**Fig. S21b**, 0.73 V *vs.* Zn^{2+}/Zn *vs.* -0.01 V *vs.* Ag/AgCl), which is caused by the H^+ concentration. Further, the voltage shift is corrected to 0.21 V based on the standard hydrogen electrode (SHE). According to the Nernst Equation, the calculated theoretical voltage shift is 0.22 V, which is close to the experimental value. This result suggests that the redox reaction participated by H^+ exists in the whole electrochemical process of the H-PNADBQ electrode. The discharge capacity of the battery in 0.05 M H_2SO_4 electrolyte is 143.8 mAh g^{-1}, indicating that H-PNADBQ can store H^+.

Calculation of potential difference based on Nernst Equation

The electrochemical reaction process of H-PNADBQ electrode in H_2SO_4 electrolyte can be expressed as below:

$$\text{H-PNADBQ} + nH^+ + ne^- \rightarrow \text{H-PNADBQ-H}_n$$

Corresponding Nernst Equation:

$$\varphi = \varphi^{\theta} + \frac{2.303RT}{nF} lg \frac{[H-PNADBQ][H^{+}]^{n}}{[H-PNADBQ-H_{n}]}$$

Where φ is electrode potential; φ^{θ} is the standard potential; R is the ideal gas constant: 8.314 J K^{-1} mol^{-1}; T is the temperature: 298.15 K; n is the electron transfer numbers; F is the Faraday constant: 96500 C mol^{-1}. The activity of solid H-PNADBQ and H-PNADBQ-H_n is considered as 1, thus the equation can be further simplified as:

$$\varphi = \varphi^{\theta} + \frac{0.0592}{n} lg[H^{+}]^{n} = \varphi^{\theta} + 0.0592\lg\,[H^{+}]$$

The pH value of 2 M $Zn(CF_3SO_3)_2$ electrolyte is about 4.7. The pH of 0.05M H2SO4 solution is about 1. Therefore, the potential difference bbetween the two electrolytes can be calculated as:

$$\Delta\varphi = 0.0529lg\frac{[H^{+}]1}{[H^{+}]2} = 0.0592lg\frac{0.1}{10^{-4.7}} = 0.22\ \text{V}$$

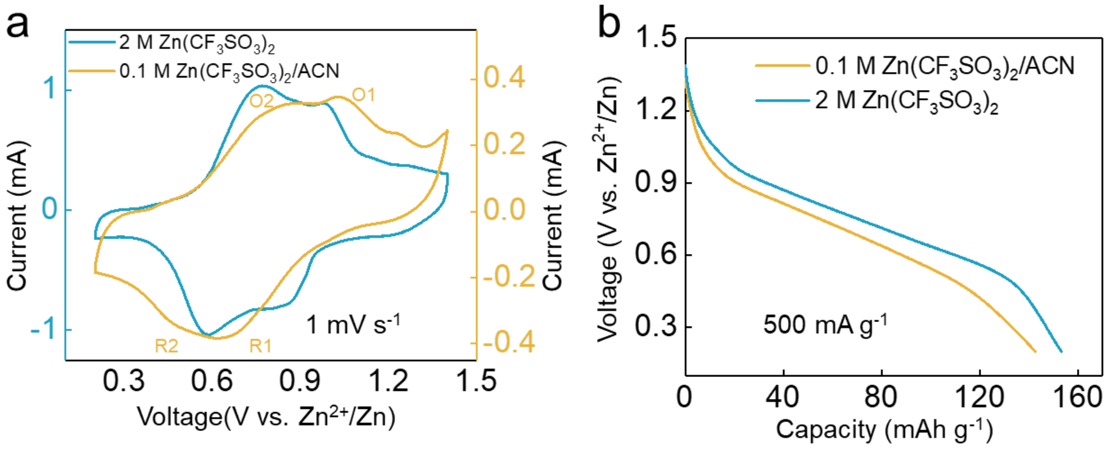

Fig. S22 a) CV curves of H-PNADBQ in different electrolytes. **b**) Charge-discharge curves of H-PNADBQ in different electrolytes (500 mA g^{-1})

Note to Figure S22:

0.1 M $Zn(CF_3SO_3)_2$ was dissolved into an acetonitrile (ACN) solution to verify the Zn^{2+}-storage ability of H-PNADBQ (**Fig. S22**). As shown in **Fig. S22a**, a pair of redox peaks in the high voltage range can be detected (O1 and R1) in the 0.1 M $Zn(CF_3SO_3)_2$/ACN electrolyte. The peaks for O2 and R2 are weak, possibly due to the restricted kinetics. The CV curves of the different electrolytes were similar in shape, and the increased voltage polarization in the 0.1 M $Zn(CF_3SO_3)_2$/ACN electrolyte could be attributed to the lower ionic conductivity. Thus, the battery in the 0.1 M $Zn(CF_3SO_3)_2$/ACN electrolyte shows a lower discharge plateau compared to the 2 M $Zn(CF_3SO_3)_2$ electrolyte (**Fig. S22b**). A high discharge capacity of 142.6 mAh g^{-1} is obtained in 0.1 M $Zn(CF_3SO_3)_2$/ACN electrolyte, indicating that H-PNADBQ has a good ability to store Zn^{2+}.

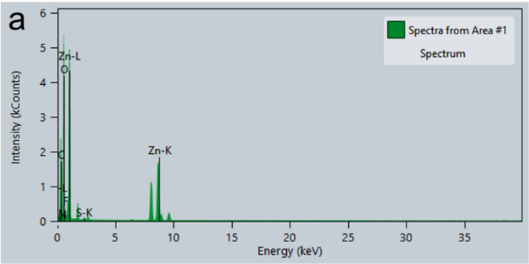

b

	Atomic Fraction (%)	Mass Fraction (%)
C	34.86	18.86
N	0.00	0.00
O	49.21	35.46
F	0.04	0.03
S	0.79	1.14
Zn	15.11	44.51

Fig. S23 a, b) EDS spectra and the elemental contents of by-products in the discharged state

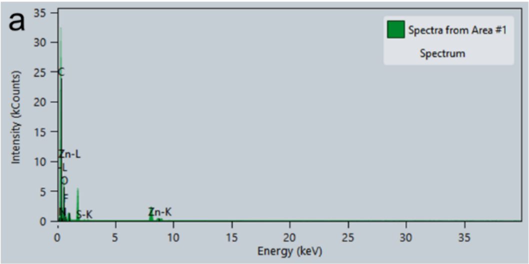

b

	Atomic Fraction (%)	Mass Fraction (%)
C	85.54	79.50
N	1.18	1.28
O	11.28	13.97
F	1.29	1.89
S	0.09	0.21
Zn	0.62	3.14

Fig. S24 a, b) EDS spectra and the elemental contents of H-PNADBQ material in the discharged state

Note to Figure S24:

To eliminate the interference of the Zn element in the $Zn_x(CF_3SO_3)_y(OH)_{2x-y}\cdot nH_2O$ by-products, the discharged H-PNADBQ electrode and by-products were completely separated by high-power ultrasonication for about 1h in the ethanol solution. Then both the by-products and individual H-PNADBQ materials can be examined under TEM. A very small amount of S elements indicates that the H-PNADBQ materials and by-products are completely separated (**Fig. S24**). Of note, the F element may originate from the binder.

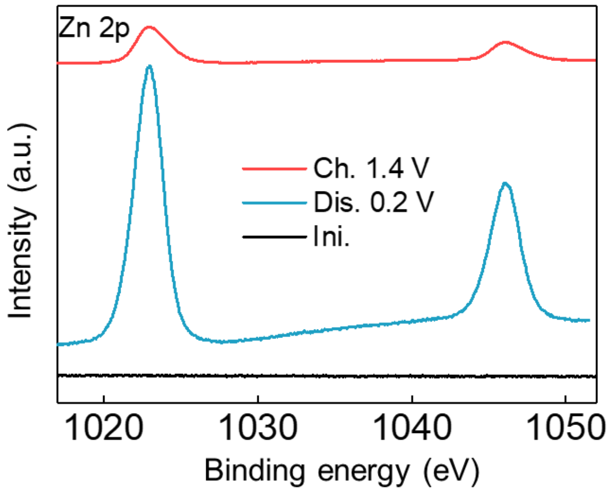

Fig. S25 XPS pattern of Zn 2p tested at different states

Supplementary References

[S1] P. Li, Y. Wang, Q. Xiong, Y. Hou, S. Yang et al., Manipulating coulombic efficiency of cathodes in aqueous zinc batteries by anion chemistry. Angew. Chem. Int. Ed. **62**, e202303292 (2023). https://dio.org/https://doi.org/10.1002/anie.202303292

[S2] Z. Cai, J. Wang, Z. Lu, R. Zhan, Y. Ou et al., Ultrafast metal electrodeposition revealed by in situ optical imaging and theoretical modeling towards fast-charging Zn battery chemistry. Angew. Chem. Int. Ed. **61**, e202116560 (2022). https://dio.org/10.1002/anie.202116560

[S3] J. Li, Q. Lin, Z. Zheng, L. Cao, W. Lv et al., How is cycle life of three-dimensional zinc metal anodes with carbon fiber backbones affected by depth of discharge and current density in zinc-ion batteries? ACS Appl. Mater. Interfaces **14**, 12323 (2022). https://dio.org/10.1021/acsami.2c00344

[S4] Z. Tie, S. Deng, H. Cao, M. Yao, Z. Niu et al., A symmetric all-organic proton battery in mild electrolyte. Angew. Chem. Int. Ed. **61**, e202115180 (2022). https://dio.org/https://doi.org/10.1002/anie.202115180

[S5] S. Zheng, D. Shi, D. Yan, Q. Wang, T. Sun et al., Orthoquinone–based covalent organic frameworks with ordered channel structures for ultrahigh performance aqueous zinc–organic batteries. Angew. Chem. Int. Ed. **61**, e202117511 (2022). https://dio.org/https://doi.org/10.1002/anie.202117511

[S6] T. Sun, W. Zhang, Q. Nian, Z. Tao, Proton-insertion dominated polymer cathode for high-performance aqueous zinc-ion battery. Chem. Eng. J. **452**, 139324 (2023). https://dio.org/10.1016/j.cej.2022.139324
